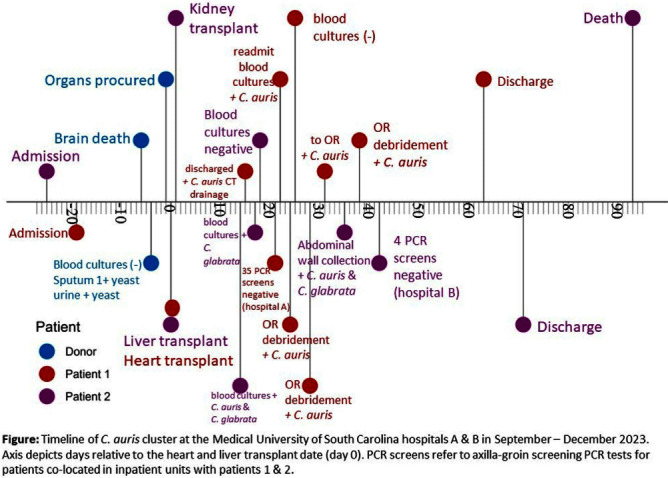# Candida auris cluster in a center with no previous infections associated with a single organ donor

**DOI:** 10.1017/ash.2024.290

**Published:** 2024-09-16

**Authors:** Evan Rivere, Eric Meissner, Alexandra Mills, Courtney Harris, Adrienne Lorek, Scott Curry

**Affiliations:** Medical University of South Carolina

## Abstract

**Background:** Candida auris is an opportunistic pathogen reported in the US since 2016. C. auris infections (CAI) are frequently healthcare-associated, but only one case of donor-derived CAI in a lung transplant recipient has been reported (PMID 28520901). We describe a cluster of two CAIs at a single center in South Carolina occurring in 2 different recipients from the same solid organ transplant donor. **Methods:** We describe two cases of invasive CAIs occurring in an academic medical center without prior CAI in Charleston, SC in October 2023. C. auris was identified using Bruker MALDI-TOF and confirmed by the state health department. **Results:** Patient 1: 40-49 year-old male underwent heart transplantation on day 19 from admission complicated by growth of C. auris on post-op day #15 from a drain. He was readmitted post-op days 22-63 with positive blood cultures for C. auris and underwent re-operation with debridement and hardware removal. C. auris pericarditis required multiple returns to the OR (Figure). He was discharged on micafungin/posaconazole with plans for long-term antifungal therapy. Patient 2: 50-59 year-old male underwent liver and kidney transplantation on day 25 from admission from the same donor as Patient 1 in a separate hospital complex. His course was complicated by possible infected biloma not amendable to drainage and C. auris/C. glabrata fungemia, which was further complicated by abdominal wall collection cultures growing C. auris on post-operative day 35 on washout. He was managed with dual micafungin/posaconazole however, he died of unrelated causes at 93 days after transplant (Figure). Investigation: The donor for both recipients was a 10-19 year-old male who suffered brain death after trauma and was hospitalized for 56 days prior to procurement in Atlanta, GA without known CAI. Airway cultures at the time of organ procurement were positive for rare Pseudomonas and light unidentified yeast of multiple morphologies; urine cultures also grew 40,000 cfu/ml un-identified yeast. Screening of 35 and 4 inpatients in units exposed to patients 1 & 2, respectively, with axilla/groin PCR was negative (Figure). A third organ recipient for this donor (kidney) at our center was identified and had negative urine fungus cultures. **Conclusions:** Despite no definitive link to a known donor infection, this cluster of CAI occurring simultaneously in 2 patients in separate hospitals/units at a single center with no known prior cases represents likely donor-derived CAI. Our experience suggests that organ procurement organizations should consider improved screening techniques for C. auris in donor cultures.

**Disclosure:** Scott Curry: Consultant- Ferring; Abbott Diagnostics- Speaker honorarium

**Figure:**